# Current Understanding and Future Research Priorities in Malignancy Associated With Inborn Errors of Immunity and DNA Repair Disorders: The Perspective of an Interdisciplinary Working Group

**DOI:** 10.3389/fimmu.2018.02912

**Published:** 2018-12-12

**Authors:** Simon Bomken, Jutte van der Werff Ten Bosch, Andishe Attarbaschi, Chris M. Bacon, Arndt Borkhardt, Kaan Boztug, Ute Fischer, Fabian Hauck, Roland P. Kuiper, Tim Lammens, Jan Loeffen, Bénédicte Neven, Qiang Pan-Hammarström, Isabella Quinti, Markus G. Seidel, Klaus Warnatz, Claudia Wehr, Arjan C. Lankester, Andrew R. Gennery

**Affiliations:** ^1^Northern Institute for Cancer Research, Newcastle University, Newcastle upon Tyne, United Kingdom; ^2^The Great North Children's Hospital, Newcastle upon Tyne Hospitals NHS Foundation Trust, Newcastle upon Tyne, United Kingdom; ^3^Department of Pediatric Hematology, Oncology and Immunology, University Hospital Brussels, Brussels, Belgium; ^4^Department of Pediatric Hematology and Oncology, St. Anna Children's Hospital, Department of Pediatrics, Medical University of Vienna, Vienna, Austria; ^5^Department of Cellular Pathology, Newcastle upon Tyne Hospitals NHS Foundation Trust, Newcastle upon Tyne, United Kingdom; ^6^Department of Pediatric Oncology, Hematology and Clinical Immunology, Medical Faculty, University Children's Hospital, Heinrich-Heine-University, Düsseldorf, Germany; ^7^Ludwig Boltzmann Institute for Rare and Undiagnosed Diseases, Vienna, Austria; ^8^CeMM Research Center for Molecular Medicine of the Austrian Academy of Sciences, Vienna, Austria; ^9^Department of Pediatrics and Adolescent Medicine, Medical University of Vienna, Vienna, Austria; ^10^Department of Pediatrics, Dr. von Hauner Children's Hospital, University Hospital, LMU Munich, Munich, Germany; ^11^Princess Máxima Center for Pediatric Oncology, Utrecht, Netherlands; ^12^Department of Pediatric Hematology-Oncology and Stem Cell Transplantation, Ghent University Hospital, Ghent, Belgium; ^13^Department of Pediatric Hematology-Immunology, Hospital Necker-Enfants Malades, Assistance Publique-Hôspitaux de Paris, INSERM, Paris, France; ^14^Department of Biosciences and Nutrition, Karolinska Institutet, Huddinge, Sweden; ^15^Department of Molecular Medicine, Sapienza University of Rome, Rome, Italy; ^16^Division of Pediatric Hematology-Oncology, Research Unit Pediatric Hematology and Immunology, Department of Pediatrics and Adolescent Medicine, Medical University Graz, Graz, Austria; ^17^Center for Chronic Immunodeficiency, Medical Center, Faculty of Medicine, Albert Ludwigs University of Freiburg, Freiburg, Germany; ^18^Section Immunology, Department of Pediatrics, Hematology and Stem Cell Transplantation, Leiden University Medical Center, Leiden, Netherlands; ^19^Institute of Cellular Medicine, Newcastle University, Newcastle upon Tyne, United Kingdom

**Keywords:** inborn error of immunity, DNA repair defect, cancer, lymphoma, EBV (Epstein-Barr virus), haematopoietic stem cell transplant, chemotherapy, screening

## Abstract

Patients with inborn errors of immunity or DNA repair defects are at significant risk of developing malignancy and this complication of their underlying condition represents a substantial cause of morbidity and mortality. Whilst this risk is increasingly well-recognized, our understanding of the causative mechanisms remains incomplete. Diagnosing cancer is challenging in the presence of underlying co-morbidities and frequently other inflammatory and lymphoproliferative processes. We lack a structured approach to management despite recognizing the competing challenges of poor response to therapy and increased risk of toxicity. Finally, clinicians need guidance on how to screen for malignancy in many of these predisposing immunodeficiencies. In order to begin to address these challenges, we brought together representatives of European Immunology and Pediatric Haemato-Oncology to define the current state of our knowledge and identify priorities for clinical and research development. We propose key developmental priorities which our two communities will need to work together to address, collaborating with colleagues around the world.

## Introduction

Patients with an inborn error of immunity (IEI) or a DNA repair disorder (DNARD) have a significantly greater risk of developing malignancies than the general population (1) with an overall relative risk varying from 1.4- to 5-fold in registry-based studies (2–5). The risk, however, varies greatly between underlying genetic conditions and within the narrow range of malignancies seen to occur, with the risk of lymphoid malignancies overall being 8–10-fold higher than age matched controls, according to a recent study from the US Immune Deficiency Network (5). Whilst many of the recognized, genetically defined IEI are very rare, disease-specific studies of the more common disorders have shown prevalences of malignancy to range from 8 to 21% in common variable immunodeficiency (6–8) to between 19 and 42% in DNARDs such as ataxia telangiectasia, Nijmegen breakage syndrome and Bloom syndrome (9–13). International collaboration, resulting in growing cohorts of patients with more recently described combined immunodeficiency disorders (14) including CD27 deficiency (15), CD70 deficiency (16), activated PI3Kδ and activated PI3Kδ2 syndromes (17, 18) has demonstrated comparable rates of malignancy, although the risk of case ascertainment bias must be considered in these very rare patient groups.

Across the spectrum of IEI/DNARD, lymphoid malignancies account for 60–70% of diagnoses and disproportionally affect children when compared to control cohorts (11, 13, 19). Teams managing patients with IEI/DNARD will recognize that both clinical and histological diagnosis of malignancy can be challenging and diagnosis may be delayed, especially in the setting of pre-existing non-malignant lymphoproliferation. Similarly, management is often complicated by an increased incidence of infectious comorbidities and severe, even life-threatening, toxicities following conventional chemotherapy or radiotherapy. These factors commonly reduce the intensity of deliverable treatment. This combination of diagnostic challenge, increased comorbidity and toxicity, and reduced therapy intensity results in an inferior outcome for patients, making malignancy a leading cause of death for this group of patients (6, 7).

**Figure 1:**
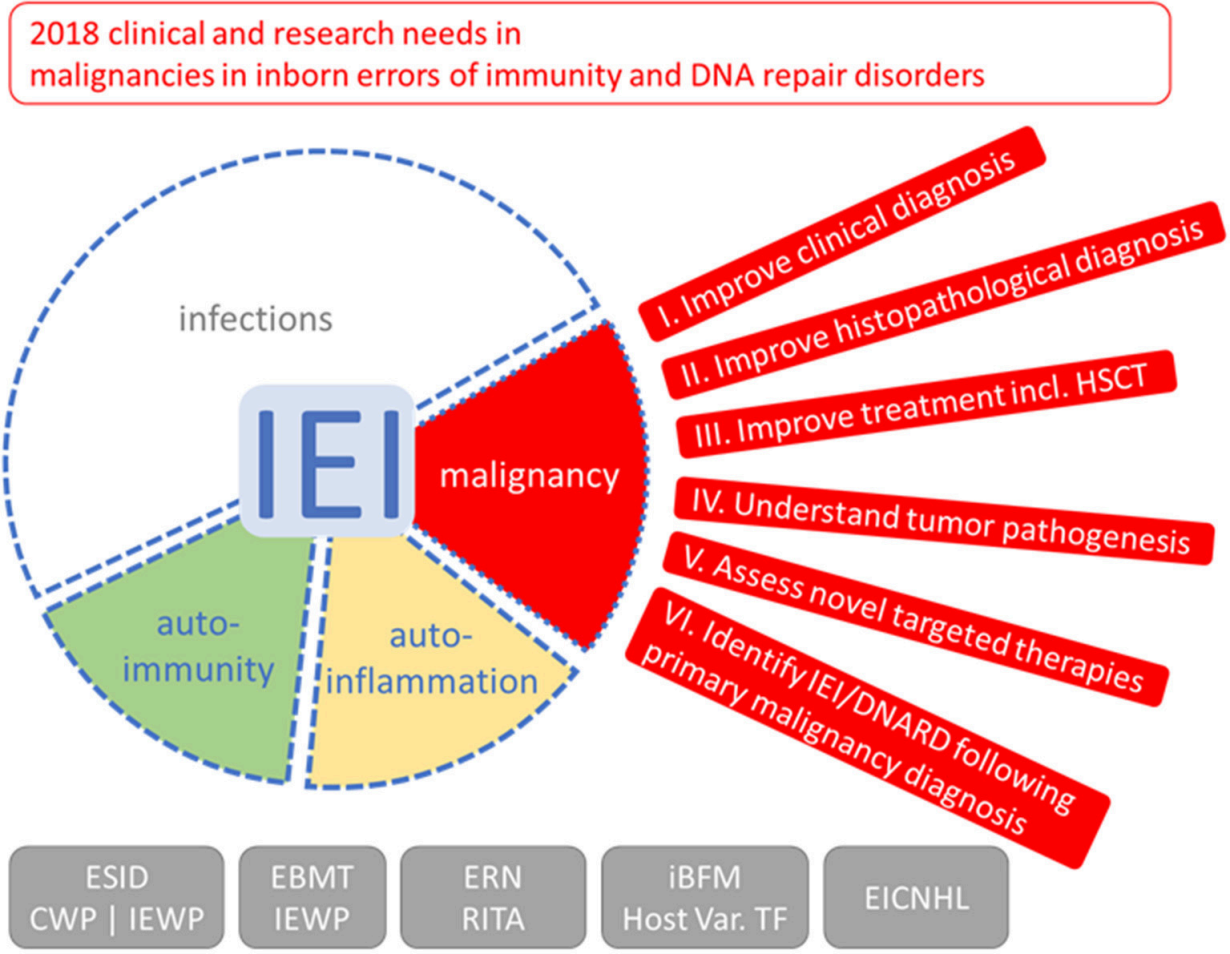
Outlook on clinical and research needs for malignancies in inborn errors of immunity (IEI) and DNA repair disorders (DNARD). Due to the increased risk and unfavorable outcome of malignancies in IEI and DNARD, the clinical working party (CWP) and the inborn errors working party (IEWP) of the European Society for Immunodeficiencies (ESID), of the European group for Blood and Marrow Transplantation (EBMT), the European Reference Network on Immunodeficiency, Autoinflammatory and Autoimmune diseases (ERN-RITA), the host variation task force (Host Var. TF) of the international Berlin-Frankfurt-Münster (iBFM) study group, and the European Intergroup Collaboration for Childhood non-Hodgkin Lymphoma (EICNHL) formulated six topics with needs and tasks for clinical management and clinical, translational, and basic scientific research to increase specific knowledge and improve management of these special malignancies; see main text and boxes for more detailed lists. The red segment depicts an eye with outlook, the spectrum of symptoms of IEI/DNARD, with infections, and, additionally, approximately 25% patients having autoimmune or autoinflammatory symptoms (20) and 4–25% of patients suffering from malignancies (21), is shown with blurred borders indicating that they are not mutually exclusive.

To improve the management of malignancy in patients affected by IEI/DNARD, we brought together a working group of representatives from both immunology and lymphoid malignancy fields to define the current state of knowledge and identify priorities for research, focusing predominantly on lymphoid malignancy (Figure 1). We present here our experience of diagnosing and managing these conditions, with a focus on the unresolved challenges faced when caring for this complex patient group. We discuss how our limited understanding of the molecular basis of oncogenesis must be expanded in order to drive the development of improved diagnostics and more effective and less toxic targeted therapies. From this we have drawn suggested priorities to be addressed through clinical and basic research collaborations. Finally, as our two fields continue to develop an increased understanding of predisposition to malignancy, we will consider the issue of identifying an underlying IEI or DNARD in patients with malignancy as a first clinical presentation.

## Mechanisms of Oncogenesis

There is an established role for a number of infectious agents in the development of both hematological and non-hematological malignancies in sporadic as well as IEI associated cases (16, 22–24). Notably, EBV is present in a significant proportion of lymphoproliferative conditions as well as other soft-tissue tumors (25). Human papillomaviruses are common in epithelial malignancies and *Helicobacter pylori* is associated with both carcinoma and extranodal marginal zone lymphoma of the stomach. Whilst, much is still unknown about the oncogenic processes these microorganisms modulate, particularly in an IEI/DNARD background (26), EBV is known to be directly oncogenic through the LMP1 protein (27). This makes IEI patients with particular susceptibility to EBV, at a very high risk of developing cancer. Nevertheless, as the same infectious agent in two patients with the same underlying condition, even siblings with identical causative mutations, can result in development of different malignancies, causation must be more complex than solely the effect of inadequate control of infection. The additional role of the host immune system in tumor immunosurveillance, a concept supported by the effective introduction of immune checkpoint inhibitor therapies, may well be important, as is consideration of the cell-intrinsic effects of the underlying IEI/DNARD, including dysregulated cellular maturation, cell signaling, apoptosis, and DNA damage responses (21, 28, 29). A well-known example of these phenomena would be the failure of clonally expanded populations to apoptose in patients with autoimmune lymphoproliferative syndrome–ALPS (30, 31).

Of particular interest is the interplay between these processes in patients with an underlying DNARD. For these patients, a number of potentially oncogenic or selective pressures accumulate, notably (i) DNA damage, especially during attempted B- and T-cell receptor rearrangement, immunoglobulin class switching and somatic hypermutation, (ii) reduced immune repertoire affecting both infectious and tumor immunosurveillance and (iii) dysregulated immune development with potential for (pre-)malignant clonal selection. This is highlighted by the substantially lower rate of tumor EBV carriage in B lymphoid proliferations in patients with ataxia telangiectasia compared to patients with other IEI (11). The relative contributions of these potential oncogenic factors are still not well-understood. Although our view of cancer susceptibility in IEI is evolving, much more in depth research will be required to define the relative importance of each of these potential causative mechanisms (32).

Currently, the cytogenetic and molecular genetic basis of malignancy in patients with IEI/DNARD is incompletely defined, despite the massive increase in genome-wide sequencing and whole transcriptome technologies over the last 15 years. In part this is due to the rarity of the individual underlying patient cohorts. However, there is also a lack of biobanked diagnostic material of sufficient quality to allow the use of advanced molecular techniques. This can, at least in part, be overcome by increasing awareness within immunology and oncology communities, but collaborative patterns of working will also accelerate progress in this area (Box 1). Addressing these challenges is important, as an improved understanding of the molecular drivers of IEI-associated malignancy will have significant benefits for patients. Firstly, mechanistic understanding, exemplified by the studies of genomic instability in acute lymphoblastic leukemia (ALL) in patients with ataxia telangiectasia (33), will elucidate causative mechanisms. Secondly, it will allow comparison with sporadic disease, driving rational selection of molecularly targeted therapies which have been developed for those counterpart conditions.

Box 1 **Developmental priorities for our understanding of oncogenesis.**

- Form collaborative networks to provide critical number of tumors and matched constitutional DNA from each defined IEI/DNARD cohort for analysis.
- Establish prospective tumor biobanks with storage of high quality diagnostic material, suitable for development of advanced diagnostic (omic) techniques.
- Develop cytogenetic and molecular biological techniques to allow analysis of historic archived material such as sequencing from formalin fixed paraffin embedded material.
- Associate new molecular knowledge with development of histopathological techniques to aid diagnosis and prognostication.

## Making a Diagnosis of Malignancy in IEI/DNARD

Despite an increased awareness of the risk of malignancy in patients with an underlying IEI or DNARD, making a definitive diagnosis can be challenging (Box 2). Many patients have complex co-morbidities, including inflammatory and infectious conditions often involving atypical organisms, non-neoplastic lymphoproliferation and bone marrow dysfunction. Each of these may mimic a developing malignancy clinically, radiologically or histopathologically. These uncertainties can result in a substantial psychological impact on the patient and family, a factor which must be borne in mind during the diagnostic process.

Box 2 **Developmental priorities for improving diagnosis and classification of malignancies.**

- Improve awareness amongst both immunologists and hematologist/oncologists of the increased risk of malignancy in particular patients with IEI/DNARD.
- Improve diagnostic and prognostic biomarkers with investigation and validation of developing technologies where appropriate.
- Prospective study of malignancies in defined cohorts of IEI/DNARD to include pathological and molecular characterization.
- Consensus diagnostic classification with integration of molecular pathological features with consideration of current and future targeted therapies.
- Develop provisional recommendations for screening cohorts at risk with studies to validate the impact of screening, including the impact on affected and unaffected family members.

Whilst radiological imaging can define the location and some features of pathology, current imaging techniques, even advanced techniques such as diffusion weighted imaging, MR spectroscopy and PET/CT, are not capable of definitive differentiation of malignant from non-malignant lesions. Furthermore, for those patients with a DNARD, there is a strong rationale for minimizing the use of ionizing radiation, resulting in understandable hesitance to undertake radiological investigations. However, in order to prevent excess mortality from delayed diagnosis it is important that alternative imaging modalities or limited exposures are used when clinical concerns of a possible malignancy exist.

Similar challenges exist for the majority of existing biomarkers. Very few blood or urine-based investigations are considered adequate for diagnosis of malignancy, with human chorionic gonadotrophin (HCG), alpha-fetoprotein (AFP), urine catecholamines and neurone specific enolase (NSE) being notable exceptions. Whilst these examples are equally valid in patients with IEI, they are infrequently relevant in diagnosis of malignancy in these patients. Cytomorphological and flow cytometric analysis of peripheral blood, bone marrow or effusions can be diagnostic in hematological malignancies, including lymphoblastic lymphomas and Burkitt lymphoma, but is commonly more complex than in patients without an underlying IEI. Other markers of lymphoproliferation, including lactate dehydrogenase, β2-microglobulin, IgM, oligoclonal/monoclonal immunoglobulin bands and serum free light chains, are commonly measured but we lack evidence to guide their use in supporting diagnosis. They may, however, have a role to play in monitoring disease following therapy. Quantitative analysis of EBV in the peripheral blood is also commonly performed, both during the diagnostic process and as a marker of response to therapy. Newer technologies developing the value of so-called liquid biopsies, such as identification of circulating tumor cells and analysis of cell-free tumor DNA, may offer the potential for a more integrative diagnostic test, sampling the “whole” patient, rather than a single lesion and identifying mutations driving malignant transformation (34). To date, these remain in early clinical development in mainstream oncology and have no established role in patients with IEI/DNARD.

Histopathology remains the cornerstone of diagnosing malignancy. However, even this gold standard approach can be challenging in patients with IEI, particularly when investigating for possible lymphoid malignancy. Firstly, judging when to perform an, often invasive, diagnostic investigation requires careful multidisciplinary discussion. Targeting the lesion with the highest likelihood of being diagnostic is a further challenge. If clinically appropriate, a surgical biopsy providing sufficient material for assessment of tissue architecture and ancillary diagnostic techniques is preferred over a needle core biopsy, but even when high quality material is obtained, histological diagnosis is often difficult. Many IEI are associated with non-neoplastic lymphoproliferations which may have specific characteristic features but together constitute a broad spectrum of processes, either inflammatory in etiology or resulting directly from the underlying genetic defect. These lesions may precede or co-exist with lymphoid malignancies and, in many settings, diagnostic boundaries between non-neoplastic and neoplastic lesions are ill-defined and difficult to apply. Existing molecular techniques to assess lymphocyte clonality may aid diagnosis, but these alone cannot provide diagnostic certainty—clonal B-cell and T-cell proliferations falling short of malignancy are not uncommon in IEI (35). Some polyclonal proliferations can be clinically aggressive and some clonal lymphomas respond to immunomodulatory therapies better than to cytotoxic chemotherapy (36). Clonal cytogenetic abnormalities may even occasionally be detected in immunodeficiency-associated lymphoid hyperplasias (37), raising the question of how to define and assess malignant transformation. In the future, personalized genomic medicine may address these challenges, but this must be established on the basis of further detailed research. Integrating these newer sources of information and deciphering diagnostic patterns will require a reference network of specialist pathologists, supported by hematologists and immunologists.

Lymphomas are the most common malignancy associated with IEI (4, 5). In many cases these are immunohistologically similar to lymphomas in immunocompetent people and can be readily diagnosed according to the WHO Classification of Tumors of Haematopoietic and Lymphoid Tissues (38). However, other lymphoproliferative disorders arising in IEI/DNARD patients are not as easily classified and the WHO Classification does not provide an explicit framework applicable to the full range of IEI/DNARD-associated tumors as it does for the post-transplant lymphoproliferative disorders (PTLD). Nevertheless, as highlighted at a recent Workshop of the Society for Hematopathology/European Association of Haematopathology (39–41), the spectrum of lymphoproliferative disorders is in many ways similar across different immunodeficiency settings including IEI and PTLD, prompting the Workshop Panel recently to propose a unifying three-part nomenclature incorporating the histopathological name of the lesion (e.g., hyperplasia, polymorphic proliferation, lymphoma classified according to the WHO Classification), any viral association, and the underlying immunodeficiency background (42). This proposal has merit in facilitating clinical and biological comparison between related lesions in conceptually similar contexts but the tumor heterogeneity seen across individual patients with the myriad IEI now recognized remains incompletely defined.

Underpinning this heterogeneity is an almost completely unknown genomic landscape that is likely to be different between immunocompromised and immunocompetent individuals, and between individuals with different IEIs, but which must be incorporated into any future pathological schema. The importance of this is clear. With the increasing availability of targeted therapies, both small molecular inhibitors and immunotherapies, understanding the underlying molecular biology of individual diseases is a clinical imperative.

One final, but important, consideration is that of screening for malignancy in patients with known predisposing conditions. This is a very challenging area, with the need to take account of factors including level of risk, location of predominant malignancies and natural history of those malignancies. In essence, each underlying IEI/DNARD must be considered separately, in keeping with a number of existing examples (43, 44), with individual validation of the benefit of screening. This will require the inclusion of large cohorts of patients with proven IEI with analysis of the incidence of all malignancies in each cohort. Despite these challenges, there may be significant benefit to early detection of malignancy, with the potential to limit treatment intensity and therefore restrict the consequent toxicity.

## Management of Malignancy in IEI/DNARD

In the majority of patients with an underlying IEI/DNARD who develop cancer, treatment should be offered with the intention of curing them of their malignancy. However, managing such a patient must balance offering potentially curative therapy with the risk of severe and even life-threatening toxicity (Box 3). This includes not only an increased risk and severity of expected toxicities, such as haemorrhagic cystitis following cyclophosphamide in patients with DNARD, but also unexpected toxicities such as cardiotoxicity and hepatotoxicity as well as deterioration of pre-existing comorbidities, especially of renal and pulmonary function. Examples of excess toxicities are well-documented in many case reports and series (11, 45–48). However, what is less well-appreciated from the literature is the variability in toxicity, even within a single disease cohort, a rare example of which has been summarized for patients with Nijmegen Breakage syndrome (49). One approach which therefore is commonly taken in IEI/DNARD is to apply an initial dose reduction strategy. This allows for subsequent dose escalation in patients not showing severe toxicity, as confirmed by frequent monitoring investigations (13, 47, 50). An alternative approach is the delivery of full-dose chemotherapy, but with increased intervals between cycles to allow optimal recovery from toxicity. Finally, protocol substitutions may be considered, with alternative agents being used in certain circumstances such as the substitution of topoisomerase inhibitors in DNARD patients. Specifically introducing novel, non-genotoxic therapies is a very attractive concept and is discussed further below. The risk is that substitution with less intensive or alternative therapies may result in under treatment and failure to achieve the primary aim, which is the cure of malignancy. Equally, whilst targeted therapies are hoped to carry less risk of toxicity, there is little clinical experience with anything other than rituximab to support this to date.

Box 3 **Developmental priorities for the treatment of malignancy including HSCT.**

- Improve understanding of cancer therapy-associated toxicities, including predictive biomarkers for toxicity
- Develop consensus guidelines for initial therapy and dose modification, dependent on underlying IEI/DNARD.
- Prospective studies addressing the timing and delivery of HSCT dependant on underlying IEI/DNARD.
- Prospective studies of the true burden of therapy on subsequent quality of life and life expectancy to guide treatment strategy decision making.
- Develop guidelines for supportive care.

Many IEI and some examples of DNARD are suitable for allogeneic haematopoietic stem cell transplantation (HSCT) and this may form a core component of the management of their underlying condition. Many centers would consider HSCT in first remission as a consolidative treatment following cytoreductive chemotherapy, with the added benefit of addressing the underlying predisposition disorder. However, there are currently a number of key unanswered questions in this area, including the state of response required prior to HSCT (partial remission vs. complete remission), optimal conditioning strategy (myeloablative vs. reduced intensity), value as salvage for refractory/recurrent malignancy and the potentially increased risk of secondary, second primary or relapsed malignancy post-transplant.

For those IEI/DNARD where transplantation is not immediately indicated, pre-emptive HSCT might be suitable for patients with a particularly high risk of developing a malignancy. This approach will require improved risk stratification and development of appropriate biomarkers. Furthermore, prolonged follow-up is required to validate outcomes of children whose risk of pediatric malignancy is reduced, but who then may continue to be at risk of other disease manifestations including an adult cancer spectrum. This is particularly true for children with DNARD (51) who, despite the use of modified conditioning regimens, may be at substantial ongoing risk of complications including non-haematopoietic malignancy.

In addition to chemotherapy, which provides the current mainstay of malignancy therapy, and HSCT, a core component of curing underlying IEI, patients require an intensive package of supportive care measures, both to allow maximal intensity of treatment delivery and to ensure minimal deterioration in baseline organ function. Dependent on the specific underlying IEI/DNARD, particular attention should be paid to surveillance for and management of infection, with many clinicians opting to keep a patient hospitalized for the duration of their cancer treatment. Upgraded supportive care strategies include more intensive immunological monitoring and wide-ranging microbiological diagnostics as well as consideration of supportive intravenous immunoglobulin, prophylactic antibiotics and haematopoietic growth factors. Episodes of presumed or confirmed infections require aggressive combinatorial broad spectrum antimicrobials, as with all patients receiving cytotoxic chemotherapy, but the duration of therapy may need to be extended and obligate bactericidal agents may be warranted. Particular attention must be paid to patients' nutrition to maximize recovery as well as to maintain intestinal integrity, minimizing translocation of intestinal organisms. Physiotherapy involvement is critical to reduce the risk of long-term respiratory deterioration from infection and to rapidly identify and manage musculoskeletal and neurological complications. Finally, clinical psychology support provides essential support for patients and families performing complex joint decision making which must address the balances of risks and benefits described above.

## Novel Therapies

The significant burden of toxicity seen in patients with IEI/DNARD following treatment with, particularly genotoxic, chemotherapy argues strongly in favor of the investigation of alternative anti-cancer treatment strategies. Existing alternatives, developed for the mainstream oncology market, rely on a range of effector mechanisms which would need to be carefully evaluated in each underlying IEI. This will be particularly important for the immune modulating therapies including checkpoint inhibitors and CAR-T cell therapies (Table 1). This presents substantial challenges, not least the relative infrequency of cancer diagnoses in patients with rare predisposing conditions. Additionally, the complex combinations of pre-existing co-morbidities would make defining tolerability very difficult in an acceptable early phase trial setting. Conducting unbiased clinical trials of novel therapies in specific patient cohorts will therefore be restricted to very few conditions.

**Table 1:** Examples of novel targeted therapeutics with potential application in IEI/DNARD associated malignancy.

**Class of agent**	**Clinical example**	**Target**	**Mechanism of action**	**Relevance in IEI/DNARD**	
Monoclonal antibody	Rituximab Alemtuzumab Daratumumab Trastuzumab Cetuximab	CD20 CD52 CD38 Her2 EGFR	Direct and indirect cellular toxicity through activation of immune targeting Competitive binding of cell surface molecule and ADCC
Antibody-drug conjugate	Brentuximab vendotin Inotuzumab ozogamicin	CD30 CD22	Targeted delivery of cytotoxic drug	No residual immune function required
Bi-specific T-cell engaging antibody	Blinatumomab	CD19-CD3	Antigen directed T cell targeting	Require functioning cytotoxic T cells
Immune checkpoint inhibitor	Nivolumab Atezolizumab	PD-1 PD-L1	Inhibit negative regulation of T cell activation	Activity may correlate with hypermutant tumors, common in CMMRD. Require functioning cytotoxic T cell
CAR-T cells	Tisagenlecleucel	CD19	Autologous T-cells expressing chimeric T-cell receptor	Requires autologous T-cell harvest – optimal efficiency likely only in functionally normal T-cells. Allogeneic options in development
Small molecule inhibitor	Ibrutinib/Acalabrutinib Idelalisib Everolimus Trametinib Crizotinib	BTK PI3Kδ mTOR MEK ALK	Inhibits BCR signaling Inhibits PI3K/AKT signaling Inhibits mTOR pathway signaling Inhibits MAPK/ERK signaling Inhibits ALK signaling	Under investigation in sporadic B/T cell malignancies commonly seen in IEI/DNARD.

*CMMRD, constitutional mismatch repair deficiency syndrome; TCR, T cell antigen receptor; BCR, B cell antigen receptor*.

Despite these difficulties, the potential impact of targeted drugs is substantial, as has already been seen with the anti-CD20 monoclonal antibody rituximab which has been used routinely as a single agent therapy in the management of PTLD for many years. Whilst achieving cure with single targeted agents has proven uncommon in general hematology/oncology, patients with IEI may prove more amenable to a strategy in which a targeted, low toxicity drug provides cytoreductive therapy prior to curative HSCT. This would still require a detailed understanding of the driving oncogenic mechanisms, biomarkers predicting response to therapy and potential mechanisms of resistance (Box 4). Developing a collaborative network of groups able to model the relevant disease process would allow for pre-clinical drug testing prior to developing a clinical strategy. Whilst randomized prospective clinical trials may be unrealistic, the need to investigate alternative therapies in these very high risk patient groups argues in favor of developing common treatment strategies with structured prospective collection of toxicity and outcome data within specific underlying IEI/DNARD cohorts.

Box 4 **Priorities for assessment and application of novel targeted therapies.**

- Improve molecular biological understanding of oncogenesis relevant to novel therapeutic approaches.
- Develop models of disease including genetically engineered mouse models and patient-derived xenografts to allow functional molecular investigations and therapy testing.
- Develop common and standardized approaches to dosing, including adjuvant chemotherapy, in order to generate structured case series.
- Prospective collection of immediate and long-term toxicity data.

## Screening for IEI in New Malignancy Cases

A number of studies have suggested that between 6 and 10% of all childhood cancer cases are the result of an underlying cancer predisposition syndrome (52–54). A significant proportion of these are not previously known to the family, but can be identified using screening approaches such as exome/genome family trio sequencing with recently identified complex patterns of inheritance identified (55, 56). Consequently, amongst all oncology patients there will be a proportion of patients carrying a previously undiagnosed IEI/DNARD. Given the increased risk of acute toxicity and infection as well as the need to address family counseling, especially with a perspective toward HSCT, identifying and investigating such patients presents an important part of their holistic care (Box 5).

Box 5 **Priorities for identification and investigation of suspected IEI/DNARD following cancer diagnosis.**

- Develop screening tools suitable for identification of IEI/DNARD at diagnosis of malignancy.
- Develop understanding of immune system status at presentation and under therapy in both sporadic and predisposed cases of malignancy.
- Longitudinal studies of immune reconstitution following treatment with both cytotoxic chemotherapy and immune system targeted therapies.
- Define algorithms for immunological investigations, including use of genomic sequencing.

In most centers consideration of immunological screening investigations has traditionally been based on the presence of additional clinical features including (i) a personal history of infections, co-morbidities, developmental delay or congenital abnormalities; (ii) a family history of known inherited conditions, strong infectious or cancer history or a consanguineous parental relationship; or (iii) unusual presentations of malignancy. The latter group may include rare tumors such as extranodal marginal zone lymphoma or peripheral T cell lymphoma in a child (57, 58), unusual background histopathological findings, unusual sites of disease (primary central nervous system lymphoma in a child) (59), or unusual characteristic cyto-/molecular genetics, our understanding of which is currently in its infancy (33). Having made a diagnosis of malignancy, the presence or occurrence of unusual infections, in terms of severity, organism or frequency, or of severe or unusual therapy associated toxicity may raise the suspicion of an underlying predisposition syndrome.

An alternative approach which is being used more frequently, and will soon be included in a number of international late phase oncology clinical trials, is the use of predisposition screening tools for newly diagnosed cancers, most notably in children (60, 61). These have been developed primarily as decision support tools to identify higher risk patients who should be referred to expert genetic counseling and consideration of targeted or genome wide investigation. However, specific screening for immune disorders as a causative underlying cancer predisposition mechanism is rarely included.

For patients in whom an increased concern regarding an underlying IEI/DNARD exists, determining the optimal time and approach to screening can be challenging. Standard laboratory investigations for underlying IEI/DNARD can be hard to interpret at diagnosis of malignancy as a result of the patient's general clinical state, the presence of infection and fever, and, for haematopoietic malignancies, involvement of the bone marrow. Investigation during and immediately following treatment is affected by anti-cancer therapy, including both traditional chemotherapy and agents targeting haematopoietic/lymphoid surface markers including CD20, CD22, CD30, and CD52 (Table 1). Direct assessment of the presence of an underlying disorder might involve radiosensitivity studies, DNA damage assays or, increasingly, genetic screening for known IEI/DNARD associated mutations using constitutional DNA samples. With the increasing availability of routine paired cancer/germline and family trio genome sequencing, this approach is likely to become more important and may bypass the challenges of more classical cellular and functional testing. However, implementation of genomic medicine at the clinical level will require dedicated analyses to be developed and probably more widespread training in interpretation and counseling to allow for immediate treatment stratification by clinicians.

## Conclusion

Despite a growing awareness of the increased risk of malignancy in people with IEI/DNARD, the diagnosis and management of these patients remain poorly understood and thus challenging and are frequently based on correlation with sporadic malignancies rather than dedicated IEI/DNARD specific guidance. In order to improve this situation, diagnostic and clinical teams caring for patients with IEI/DNARD will need to work in collaborative groups and international networks in order to bring together sufficient cases, experience and understanding. This first meeting of ESID, EBMT, RITA, iBFM, and EICNHL represents an example of such a collaborative initiative. Having developed this (non-exhaustive) list of developmental priorities, it is now important to broaden collaborations in order to maximize achievements that will allow improvement in patient care and outcomes.

## Author Contributions

All authors developed the concept of the current position and edited the manuscript. SB wrote the manuscript. MS created the graphic figure.

### Conflict of Interest Statement

The authors declare that the research was conducted in the absence of any commercial or financial relationships that could be construed as a potential conflict of interest.

## References

[1] Attarbaschi A Carraro E Abla O Barzilai-Birenboim S Bomken S Brugieres L. Non-Hodgkin lymphoma and pre-existing conditions: spectrum, clinical characteristics and outcome in 213 children and adolescents. Haematologica (2016) 101:1581–91. 10.3324/haematol.2016.147116 27515251 PMC5479624

[2] Kinlen LJ Webster AD Bird AG Haile R Peto J Soothill JF Prospective study of cancer in patients with hypogammaglobulinaemia. Lancet (1985) 325:263–6. 10.1016/S0140-6736(85)91037-2 2857327

[3] Mellemkjr L Hammarstrom L Andersen V Yuen J Heilmann C Barington T. Cancer risk among patients with IgA deficiency or common variable immunodeficiency and their relatives: a combined Danish and Swedish study. Clin Exp Immunol. (2002) 130:495–500. 10.1046/j.1365-2249.2002.02004.x 12452841 PMC1906562

[4] Jonkman-Berk BM van den Berg JM ten Berge IJM Bredius RGM Driessen GJ Dalm VASH. Primary immunodeficiencies in the Netherlands: national patient data demonstrate the increased risk of malignancy. Clin Immunol. (2015) 156:154–62. 10.1016/j.clim.2014.10.003 25451158

[5] Mayor PC Eng KH Singel KL Abrams SI Odunsi K Moysich KB. Cancer in primary immunodeficiency diseases: cancer incidence in the United States Immune Deficiency Network Registry. J Allergy Clin Immunol. (2018) 141:1028–35. 10.1016/j.jaci.2017.05.024 28606585 PMC5723251

[6] Resnick ES Moshier EL Godbold JH Cunningham-Rundles C. Morbidity and mortality in common variable immune deficiency over 4 decades. Blood (2012) 119:1650–7. 10.1182/blood-2011-09-377945 22180439 PMC3286343

[7] Quinti I Agostini C Tabolli S Brunetti G Cinetto F Pecoraro A. Malignancies are the major cause of death in patients with adult onset common variable immunodeficiency. Blood (2012) 120:1953–4. 10.1182/blood-2012-05-431064 22936739

[8] Gathmann B Mahlaoui N Gérard L Oksenhendler E Warnatz K Schulze I. Clinical picture and treatment of 2212 patients with common variable immunodeficiency. J Allergy Clin Immunol. (2014) 134:116–26. 10.1016/j.jaci.2013.12.1077 24582312

[9] Morrell D Cromartie E Swift M. Mortality and cancer incidence in 263 patients with Ataxia Telangiectasia. J Natl Cancer Inst (1968) 77:89–92 3459930

[10] German J. Bloom's syndrome. XX. The first 100 cancers. Cancer Genet Cytogenet. (1997) 93:100–6. 9062585 10.1016/s0165-4608(96)00336-6

[11] Suarez F Mahlaoui N Canioni D Andriamanga C Dubois d'Enghien C Brousse N. Incidence, presentation, and prognosis of malignancies in ataxia-telangiectasia: a report from the French national registry of primary immune deficiencies. J Clin Oncol (2015) 33:202–8. 10.1200/JCO.2014.56.5101 25488969

[12] Wolska-Kuśnierz B Gregorek H Chrzanowska K Piatosa B Pietrucha B Heropolitanska-Pliszka E. Nijmegen breakage syndrome: clinical and immunological features, long-term outcome and treatment options - a retrospective analysis. J Clin Immunol. (2015) 35:538–49. 10.1007/s10875-015-0186-9 26271390

[13] van Os NJH Jansen AFM van Deuren M Haraldsson A van Driel NTM Etzioni A Ataxia-telangiectasia: immunodeficiency and survival. Clin Immunol. (2017) 178:45–55. 10.1016/j.clim.2017.01.009 28126470

[14] Picard C Al-Herz W Bousfiha A Casanova JL Chatila T Conley ME. Primary immunodeficiency diseases: an update on the classification from the International Union of Immunological Societies Expert Committee for Primary Immunodeficiency 2015. J Clin Immunol. (2015) 35:696–726. 10.1007/s10875-015-0201-1 26482257 PMC4659841

[15] Salzer E Daschkey S Choo S Gombert M Santos-Valente E Ginzel S Combined immunodeficiency with life-threatening EBV-associated lymphoproliferative disorder in patients lacking functional CD27. Haematologica (2013) 38:473–8. 10.3324/haematol.2012.068791 PMC3659923 22801960

[16] Abolhassani H Edwards ES Ikinciogullari A Jing H Borte S Buggert M. Combined immunodeficiency and Epstein-Barr virus-induced B cell malignancy in humans with inherited CD70 deficiency. J Exp Med. (2017) 214:91–106. 10.1084/jem.20160849 28011864 PMC5206499

[17] Elkaim E Neven B Bruneau J Mitsui-Sekinaka K Stanislas A Heurtier L. Clinical and immunologic phenotype associated with activated phosphoinositide 3-kinase δ syndrome 2: a cohort study. J Allergy Clin Immunol. (2016) 138:210–8. 10.1016/j.jaci.2016.03.022 27221134

[18] Coulter TI Chandra A Bacon CM Babar J Curtis J Screaton N. Clinical spectrum and features of activated phosphoinositide 3-kinase δ syndrome: a large patient cohort study. J Allergy Clin Immunol. (2017) 139:597–606. 10.1016/j.jaci.2016.06.021 27555459 PMC5292996

[19] Taylor AMR Metcalfe JA Thick J Mak YF. Leukemia and lymphoma in Ataxia Telangiectasia. Blood (1996) 87:423–38. 8555463

[20] Fischer A Provot J Jais JP Alcais A Mahlaoui N members of the CEREDIH French PID study group Autoimmune and inflammatory manifestations occur frequently in patients with primary immunodeficiencies J Allergy Clin Immunol. (2017) 140:1388–93. 10.1016/j.jaci.2016.12.978 28192146

[21] Hauck F Voss R Urban C Seidel MG. Intrinsic and extrinsic causes of malignancies in patients with primary immunodeficiency disorders. J Allergy Clin Immunol. (2018) 141:59–68. 10.1016/j.jaci.2017.06.009 28669558

[22] Parsonnet J Friedman GD Vandersteen DP Chang Y Vogelman JH Orentreich N. Helicobacter pylori infection and the risk of gastric carcinoma. N Engl J Med (1991) 325:1127–31. 10.1056/NEJM199110173251603 1891020

[23] Wheat WH Cool CD Morimoto Y Rai PR Kirkpatrick CH Lindenbaum BA. Possible role of human herpesvirus 8 in the lymphoproliferative disorders in common variable immunodeficiency. J Exp Med. (2005) 202:479–84. 10.1084/jem.20050381 16103407 PMC2212861

[24] Moore PS Chang Y. Why do viruses cause cancer? Highlights of the first century of human tumour virology. Nat Rev Cancer (2010) 10:878–89. 10.1038/nrc2961 21102637 PMC3718018

[25] Dekate J Chetty R. Epstein-Barr virus–associated smooth muscle tumor. Arch Pathol Lab Med. (2016) 140:718–22. 10.5858/arpa.2015-0120-RS 27362573

[26] Young LS Yap LF Murray PG. Epstein-Barr virus: more than 50 years old and still providing surprises. Nat Rev Cancer (2016) 16:789–802. 10.1038/nrc.2016.92 27687982

[27] Wang D Liebowitz D Kieff E. An EBV membrane protein expressed in immortalized lymphocytes transforms established rodent cells. Cell (1985) 43:831–40. 10.1016/0092-8674(85)90256-9 3000618

[28] Swann JB Smyth MJ. Immune surveillance of tumors. J Clin Invest. (2007) 117:1137–46. 10.1172/JCI31405 17476343 PMC1857231

[29] Ribatti D. The concept of immune surveillance against tumors. The first theories.Oncotarget (2017) 8:7175–80. 10.18632/oncotarget.12739 27764780 PMC5351698

[30] Straus SE Jaffe ES Puck JM Dale JK Elkon KB Rösen-Wolff A. The development of lymphomas in families with autoimmune lymphoproliferative syndrome with germline Fas mutations and defective lymphocyte apoptosis. Blood (2001) 98:194–200. 10.1182/blood.V98.1.194 11418480

[31] Poppema S Maggio E van den Berg A. Development of lymphoma in Autoimmune Lymphoproliferative Syndrome (ALPS) and its relationship to Fas gene mutations. Leuk Lymphoma (2004) 45:423–31. 10.1080/10428190310001593166 15160902

[32] Satgé D. A tumor profile in primary immune deficiencies challenges the cancer immune surveillance concept. Front Immunol. (2018) 9:1149. 10.3389/fimmu.2018.01149 29881389 PMC5976747

[33] Ratnaparkhe M Hlevnjak M Kolb T Jauch A Maass KK Devens F. Genomic profiling of acute lymphoblastic leukemia in ataxia telangiectasia patients reveals tight link between ATM mutations and chromothripsis. Leukemia (2017) 31:2048–56. 10.1038/leu.2017.55 28196983

[34] Darrah JM Herrera AF. Updates on circulating tumor DNA assessment in lymphoma. Curr Hematol Malig Rep. (2018) 13:348–55. 10.1007/s11899-018-0468-4 30136210

[35] Gompels MM Hodges E Lock RJ Angus B White H Larkin A. Lymphoproliferative disease in antibody deficiency: a multi-centre study. Clin Exp Immunol. (2003) 134:314–20. 10.1046/j.1365-2249.2003.02253.x 14616793 PMC1808874

[36] Michonneau D Petrella T Ortonne N Ingen-Housz-Oro S Franck N Barete S. Subcutaneous panniculitis-like T-cell Lymphoma: immunosuppressive drugs induce better response than polychemotherapy. Acta Derm Venereol. (2017) 97:358–64. 10.2340/00015555-2543 27722764

[37] Vakiani E Nandula SV Subramaniyam S Keller CE Alobeid B Murty VV. Cytogenetic analysis of B-cell posttransplant lymphoproliferations validates the World Health Organization classification and suggests inclusion of florid follicular hyperplasia as a precursor lesion. Hum Pathol. (2007) 38:315–25. 10.1016/j.humpath.2006.08.014 17134734

[38] Swerdlow SH Campo E Harris NL Jaffe ES Pileri SA Stein H WHO Classification of Tumours of Haematopoietic and Lymphoid Tissues. Revised 4th edn. Lyon: International Agency for Research on Cancer (2016).

[39] Natkunam Y Goodlad JR Chadburn A de Jong D Gratzinger D Chan JK. EBV-positive B-cell proliferations of varied malignant potential: 2015 SH/EAHP workshop report-Part 1. Am J Clin Pathol. (2017) 147:129–52. 10.1093/ajcp/aqw214 28395107 PMC6248636

[40] de Jong D Roemer MG Chan JK Goodlad J Gratzinger D Chadburn A. B-cell and classical Hodgkin lymphomas associated with immunodeficiency: 2015 SH/EAHP Workshop Report-Part 2. Am J Clin Pathol. (2017) 147:153–70. 10.1093/ajcp/aqw216 28395108 PMC6248547

[41] Gratzinger D Jaffe ES Chadburn A Chan JK de Jong D Goodlad JR. Primary/Congenital Immunodeficiency: 2015 SH/EAHP Workshop Report-Part 5. Am J Clin Pathol (2017) 147:204–16. 10.1093/ajcp/aqw215 28395106 PMC6248572

[42] Natkunam Y Gratzinger D Chadburn A Goodlad JR Chan JKC Said J. Immunodeficiency-associated lymphoproliferative disorders: time for a reappraisal? Blood (2018) 132:1871–78. 10.1182/blood-2018-04-842559 30082493 PMC6213318

[43] Tabori U Hansford JR Achatz MI Kratz CP Plon SE Frebourg T Clinical management and tumor surveillance recommendations of inherited mismatch repair deficiency in childhood Clin Cancer Res. (2017) 23:e32–e37. 10.1158/1078-0432.CCR-17-0574 28572265

[44] Walsh MF Chang VY Kohlmann WK Scott HS Cunniff C Bourdeaut F. Recommendations for childhood cancer screening and surveillance in DNA Repair Disorders. Clin Cancer Res. (2017) 23:e23–e31. 10.1158/1078-0432.CCR-17-0465 28572264 PMC5697784

[45] Seidemann K Tiemann M Henze G Sauerbrey A Müller S Reiter A. Therapy for non-Hodgkin lymphoma in children with primary immunodeficiency: Analysis of 19 patients from the BFM trials. Med Pediatr Oncol. (1999) 33:536–44. 10573576 10.1002/(sici)1096-911x(199912)33:6<536::aid-mpo3>3.0.co;2-z

[46] Sandoval C Swift M Hodgkin disease in ataxia-telangiectasia patients with poor outcome. Med Pediatr Oncol. (2003) 40:162–6. 10.1002/mpo.10251 12518345

[47] Sandlund JT Hudson MM Kennedy W Onciu M Kastan MB. Pilot study of modified LMB-based therapy for children with ataxia-telangiectasia and advanced stage high grade mature B-cell malignancies. Pediatr Blood Cancer (2014) 61:360–2. 10.1002/pbc.24696 23900766 PMC4254821

[48] Jastaniah W. Successful treatment of mature B-cell lymphoma with rituximab-based chemotherapy in a patient with Bloom syndrome. Pediatr Blood Cancer (2016) 64:e26385. 10.1002/pbc.26385 27966805

[49] Pastorczak A Szczepanski T Mlynarski W;International Berlin-Frankfurt-Munster (I-BFM) ALL host genetic variation working group. Clinical course and therapeutic implications for lymphoid malignancies in Nijmegen breakage syndrome. Eur J Med Genet. (2016). 59:126–32. 10.1016/j.ejmg.2016.01.007 26826318

[50] Schoenaker MH Suarez F Szczepanski T Mahlaoui N Loeffen JL. Treatment of acute leukemia in children with ataxia telangiectasia (A-T). Eur J Med Genet. (2016) 59:641–6. 10.1016/j.ejmg.2016.05.012 27238889

[51] Slack J Albert MH Balashov D Belohradsky BH Bertaina A Bleesing J;Inborn errors working party of the european society for blood and marrow transplantation and the european society for immunodeficiencies; Stem Cell Transplant for Immunodeficiencies in Europe (SCETIDE); Center for International Blood and Marrow Transplant Research; Primary Immunodeficiency Treatment Consortium. Outcome of hematopoietic cell transplantation for DNA double-strand break repair disorders. J Allergy Clin Immunol. (2017) 141:322–8. 10.1016/j.jaci.2017.02.036 28392333 PMC5632132

[52] Zhang J Walsh MF Wu G Edmonson MN Gruber TA Easton J. Germline mutations in predisposition genes in pediatric cancer. N Engl J Med. (2015) 373:2336–46. 10.1056/NEJMoa1508054 26580448 PMC4734119

[53] Grbner SN Worst BC Weischenfeldt J Buchhalter I Kleinheinz K Rudneva VA The landscape of genomic alterations across childhood cancers. Nature (2018) 555:321–7. 10.1038/nature25480 29489754

[54] Huang KL Mashl RJ Wu Y Ritter DI Wang J Oh C. Pathogenic germline variants in 10,389 adult cancers. Cell (2018) 173:355–70. 10.1016/j.cell.2018.03.039 29625052 PMC5949147

[55] Kuhlen M Borkhardt A. Trio sequencing in pediatric cancer and clinical implications. EMBO Mol Med. (2018) 10:e8641. 10.15252/emmm.201708641 29507082 PMC5887902

[56] Diets IJ Waanders E Ligtenberg MJ van Bladel DAG Kamping EJ Hoogerbrugge PM. High yield of pathogenic germline mutations causative or likely causative of the cancer phenotype in selected children with cancer. Clin Cancer Res. (2018) 24:1594–603. 10.1158/1078-0432.CCR-17-1725 29351919

[57] Ronceray L Abla O Barzilai-Birenboim S Bomken S Chiang AK Jazbec J Children and adolescents with marginal zone lymphoma have an excellent prognosis with limited chemotherapy or a watch-and-wait strategy after complete resection. Pediatr Blood Cancer (2018) 65:4 10.1002/pbc.26932 29286565

[58] Mellgren K Attarbaschi A Abla O Alexander S Bomken S Bubanska E European Intergroup for Childhood Non-Hodgkin Lymphoma (EICNHL) and the international Berlin-Frankfurt-Münster (i-BFM) Group. Non-anaplastic peripheral T cell lymphoma in children and adolescents-an international review of 143 cases. Ann Hematol. (2016) 95:1295–305. 10.1007/s00277-016-2722-y 27270301

[59] Thorer H Zimmermann M Makarova O Oschlies I Klapper W Lang P. Primary central nervous system lymphoma in children and adolescents: low relapse rate after treatment according to Non-Hodgkin-Lymphoma Berlin-Frankfurt-Münster protocols for systemic lymphoma. Haematologica (2014) 99:e238–241. 10.3324/haematol.2014.109553 25107886 PMC4222469

[60] Jongmans MC Loeffen JL Waanders E Hoogerbrugge PM Ligtenberg MJ Kuiper RP. Recognition of genetic predisposition in pediatric cancer patients: an easy-to-use selection tool. Eur J Med Genet. (2016) 59:116–25. 10.1016/j.ejmg.2016.01.008 26825391

[61] Postema FA Hopman SM de Borgie CA Hammond P Hennekam RC Merks JH. Validation of a clinical screening instrument for tumour predisposition syndromes in patients with childhood cancer (TuPS): protocol for a prospective, observational, multicentre study. BMJ Open (2017) 7:e013237. 10.1136/bmjopen-2016-013237 28110285 PMC5253556

